# Effectiveness and Safety of Etanercept in Paediatric Patients with Plaque-Type Psoriasis: Real-World Evidence

**DOI:** 10.3390/jcm13164858

**Published:** 2024-08-17

**Authors:** Joanna Narbutt, Zofia Jakubczak, Paulina Wasiewicz-Ciach, Joanna Wojtania, Katarzyna Krupa, Dorota Sobolewska-Sztychny, Magdalena Ciążyńska, Marta Kołt-Kamińska, Adam Reich, Małgorzata Skibińska, Aleksandra Lesiak

**Affiliations:** 1 Department of Dermatology, Paediatric Dermatology and Oncology, Medical University of Lodz, 90-419 Lodz, Poland; joanna.narbutt@umed.lodz.pl (J.N.); katarzyna.krupa100@gmail.com (K.K.); dorota.sobolewska-sztychny@umed.lodz.pl (D.S.-S.); malgorzata.skibinska@umed.lodz.pl (M.S.); 2 Students’ Scientific Association of Experimental, Clinical and Surgical Dermatology, Medical University of Lodz, 91-347 Lodz, Poland; zofia.jakubczak8@gmail.com (Z.J.); paulina.wasiewicz@gmail.com (P.W.-C.); joanna.wojtania@outlook.com (J.W.); 3 Laboratory of Autoinflammatory, Genetic and Rare Skin Disorders, Department of Dermatology, Pediatric Dermatology and Oncology, Medical University of Lodz, 91-347 Lodz, Poland; magdalena.ciazynska@umed.lodz.pl; 4 Department of Dermatology, Institute of Medical Sciences, Medical College of Rzeszow University, 35-055 Rzeszow, Poland; marta.kolt@o2.pl (M.K.-K.); adamandrzejreich@gmail.com (A.R.)

**Keywords:** psoriasis, etanercept, children

## Abstract

**Background**: Psoriasis is a chronic, multisystemic, inflammatory disease affecting approximately 1% of children and significantly reducing their health-related quality of life. Etanercept is a biologic fusion protein-blocking TNF-α and belongs to one of the biologics used among the children population. The purpose of this study was to assess the effectiveness and safety profile of etanercept in paediatric patients with plaque-type psoriasis. Material and methods: The outcome of the treatment was evaluated based on Psoriasis Area and Severity Index (PASI), Body Surface Area (BSA), and Children’s Dermatology Life Quality Index (CDLQI). Achievement of at least PASI75 at week 16 was assessed as an adequate response to therapy, which was the primary endpoint. **Results**: Forty-three paediatric patients were included in the study, 24 females and 19 males. The average age at inclusion into our study was 13 years. At baseline, the mean PASI score, BSA, and CDLQI were 16.3 ± 6.5, 22.3 ± 12.2%, and 17.4 ± 5.3, respectively. At week 16, 90.7% of patients achieved PASI 50, 79.1% achieved PASI 75, and 46.5% attained PASI 90. There was also a decrease in mean BSA and CDLQI values to 3.5 ± 3.8 and 5.4 ± 5.7, respectively. **Conclusions**: Etanercept proved to be effective, safe, and well-tolerated among the paediatric population with psoriasis.

## 1. Introduction

Psoriasis is a systemic, immune-mediated disease with multiple manifestations involving erythematous, scaly skin lesions localised mainly around the knees, elbows, scalp, and trunk, with frequent nail and joint involvement [1,2]. Plaque-type psoriasis is the most common form of psoriasis, characterised by well-defined, round or oval plaques differing in their size [1,3]. Psoriasis, particularly moderate-to-severe psoriasis, is associated with a number of comorbidities, such as cardiovascular diseases, psoriatic arthritis, lymphoma, inflammatory bowel disease, depression, and anxiety [1,4,5]. The prevalence of psoriasis among children in Europe is about 0.18–0.55% in paediatric patients aged 0–9 years and 0.83–1.37% in adolescents aged 10–19 years [6,7,8]. Furthermore, psoriasis constitutes 4% of all dermatological problems among children under 16 years of age [9]. This chronic, immune-mediated inflammatory skin disease is significantly reducing children’s health-related quality of life (QoL), causing social isolation and problems at school [10].

Therefore, effective treatment is essential for paediatric patients to prevent the development of serious comorbidities and stigmatisation. Topical drugs are used as monotherapy in children with mild forms of psoriasis (with Body Surface Area (BSA) < 10% and Psoriasis Area and Severity Index (PASI) < 10 points) and as an adjunct therapy to phototherapy and systemic therapies among children with more severe lesions. Local treatment of psoriasis is planned individually, depending on the child’s age, extent of lesions, their location, the socio-economic situation of the family, and the patient’s lifestyle. The patients’ and their guardians’ ability to adapt to the principles of treatment is also crucial. To control the disease and achieve clinical remission in children with moderate-to-severe psoriasis (PASI ≥ 10 points and/or BSA ≥ 10% and/or Children Dermatology Life Quality Index (CDLQI) ≥ 10 points), phototherapy and systemic treatments are used. Systemic drugs are to ensure improvement, with the lowest effective dose and minimum side effects. As per Polish guidelines, a good therapeutic effect (achievement of PASI90 or PASI75 and CDLQI ≤ 5 points) allows for the continuation of the therapy. Analogously, an unsatisfactory result of the conducted treatment (PASI reduction < 75% or achievement of PASI75 but CDLQI > 5 points) creates the necessity of its modification [11].

Frequent difficulties with the management of psoriatic symptoms and prevention of flares during conventional therapies make biological treatment a convenient and safe option for affected patients, which could even be considered as the first-line treatment in the paediatric population with psoriasis [12]. The Polish B.47 drug programme [13] provides reimbursed biological treatment for both adult and paediatric patients with moderate-to-severe plaque psoriasis. The treatment is available for patients fulfilling the inclusion criteria. One of the available drugs for plaque-type psoriasis is etanercept, which, for many years, was the only reimbursed biologics for paediatric patients with psoriasis in Poland. Etanercept is a fusion protein within the Tumour Necrosis Factor (TNF) blockers. TNF is a cytokine that can bind to TNF receptor 1 (TNFR1) or TNF receptor 2 (TNFR2) and is a key factor in mediating inflammation in psoriasis [14]. Etanercept works by blocking the effects of TNF-alpha, whose level is elevated in psoriasis and multiple other immune-mediated diseases, such as Crohn’s disease, ulcerative colitis, rheumatoid arthritis, psoriatic arthritis, and ankylosing spondylitis [14]. Etanercept was the first drug investigated in a randomised controlled trial versus placebo for the treatment of psoriasis among the paediatric population [15]. Additionally, it was the first biological medicine licensed for the treatment of chronic severe plaque psoriasis in children aged ≥ 6 years in Europe and ≥4 years in the USA [16,17].

Etanercept was the only medicine used in the B.47 drug programme among the children population until the year 2023. Currently, the programme also includes adalimumab, ustekinumab, ixekizumab, and secukinumab [13].

Evidence for effectiveness and safety of biological systemic treatments in moderate-to-severe psoriasis in children and adolescents is still unsatisfactory. The purpose of this study was to assess the effectiveness–safety profile of etanercept treatment in paediatric patients with moderate-to-severe plaque psoriasis as well as to raise the awareness of the importance of more frequent implementation of biological treatment into a patient’s therapeutic process as a safe and convenient therapeutic alternative. Our study is the largest observational study assessing treatment with etanercept among children in Poland.

## 2. Materials and Methods

The study was conducted in the Department of Dermatology, Paediatric Dermatology and Oncology at the Medical University of Lodz in Łódź, Poland and in the Department of Dermatology in Rzeszów, Poland. Etanercept was administered to the first patient in January 2020. Our study was performed in accordance with the Helsinki Declaration of 1964, along with its later amendments, and received the consent of the Bioethics Committee at the Medical University of Lodz.

Psoriasis severity was assessed based on the following: PASI, BSA, and CDLQI. All of them provided a valid evaluation of psoriasis. Inclusion criteria were as follows: moderate-to-severe psoriasis; age between 6 and 18 years; PASI, BSA, and CDLQI scores of at least 10 points; and/or lesions localised in special areas, including face, scalp, nails, genitalia, palms, and soles. Patients were enrolled to the study if they failed to respond to systemic or topical therapy or had contraindications to classic systemic anti-psoriatic therapy.

Treatment was stopped if patients did not respond to the treatment at week 16 (adequate response—achievement of PASI75 or PASI50 and concomitant decrease in CDLQI by at least 5 points). Treatment cessation was also performed if we noted loss of response to the drug defined as PASI, BSA, and CDLQI scores more than 10 at each of two consecutive follow-up visits or if the patient experienced a serious adverse event (AEs) considered as contraindication to the continuation of therapy.

### 2.1. Medications

During the study, the patients received etanercept at a dose of 0.8 mg/kg (up to maximum of 50 mg per dose), as recommended in the Summary of Product Characteristics [18]. The drug was administered subcutaneously once weekly. The patients were allowed to use topical drugs during the study.

### 2.2. Endpoints

Follow-up visits were performed 2 (±30 days) and 4 months (±30 days) after the first administration of etanercept. Subsequently, PASI, BSA, and CDLQI were measured at weeks 4, 16, 40, and 64 and then once every 6 months or more frequently.

The primary endpoint of the study was 75% or higher improvement in PASI (PASI75) at week 16. PASI 90 and PASI 50 were the secondary endpoints.

PASI90 (Psoriasis Area and Severity Index 90) refers to a 90% reduction in the Psoriasis Area and Severity Index (PASI) score from baseline levels. This endpoint signifies an advanced level of therapeutic efficacy, illustrating an extensive amelioration in the clinical parameters of psoriasis, including reductions in erythema, scaling, and induration. A PASI90 response represents an exceptional degree of improvement, with the PASI score decreasing by at least 90% from the original baseline measurement.

PASI75 (Psoriasis Area and Severity Index 75) denotes a reduction of 75% in the Psoriasis Area and Severity Index (PASI) score from the baseline measurement. The PASI score is a quantitative tool used to assess the clinical severity and extent of psoriasis by evaluating the area of skin affected and the degree of erythema, scaling, and induration. Achieving a PASI75 response indicates a substantial therapeutic effect, reflecting a significant decrease in the overall PASI score, with an improvement threshold of 75% from the initial baseline.

PASI50 (Psoriasis Area and Severity Index 50) indicates a 50% reduction in the Psoriasis Area and Severity Index (PASI) score from the baseline. This measure reflects a moderate degree of therapeutic improvement in the severity and extent of psoriasis. A PASI50 response demonstrates a significant, albeit less pronounced, reduction in psoriasis symptoms, with the PASI score being reduced by at least 50% compared to the initial baseline assessment [19].

Laboratory tests performed prior to initiate therapy with etanercept included the following: baseline blood tests such as a complete blood count, liver function tests with alanine transaminase (ALT) and aspartate transaminase (AST), and a creatinine and C-reactive protein (CRP) test. Patients had to be screened for latent infections: tuberculosis (TB), hepatitis B virus (HBV) infection, and hepatitis C virus (HCV) infection. To detect potential HIV infection, HIV antigen and antibody tests were performed. Additionally, an electrocardiogram and chest radiogram were carried out. Patients were weighed before their first injection. Percentiles were calculated based on the calculator OLA and OLAF and growth charts established by the World Health Organisation [20]. Laboratory tests including a complete blood count, liver function tests, a creatinine blood test, and C-reactive protein level were performed at weeks 8 and 16 after treatment initiation, and afterwards, once every six months.

The safety profile was evaluated based on the incidence of adverse events and abnormalities in the blood test results. All patients were monitored for the most common adverse events: injection-site reactions, respiratory infections, nasopharyngitis, headache, acne, cellulitis, and of course, opportunistic infections. During the follow-up appointments, laboratory assessments were conducted, including a complete blood count, C-reactive protein (CRP) quantification, alanine transaminase (ALT) assay, aspartate transaminase (AST) assay, and a creatinine determination.

### 2.3. Statistical Analysis

Effectiveness and safety analyses at week 16 included all patients who achieved two control points (weeks 4 and 16). Patients were enrolled into our study on different dates. As a result, the number of patients varies between specific time points.

## 3. Results

### 3.1. Patients Characteristics

**Figure 1:**
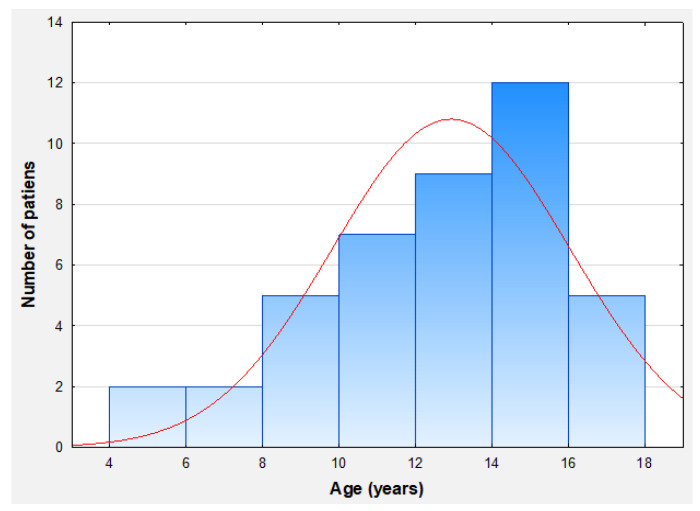
Age distribution of analysed patients.

A total of 43 patients with moderate-to-severe plaque psoriasis were recruited into the study. Of note, this is the largest observational study in Poland focusing on the paediatric population affected by psoriasis treated with etanercept. Females constituted the majority of the study group (24 out of 43 patients; 55.8%). The age of the patients ranged from 6 to less than 18 years old, with the mean age at inclusion of 13 years. The detailed age distribution is depicted in Figure 1.

The mean weight of the patients was 66 kg, which corresponded to the 75th percentile on the growth charts. The mean body weight of females and males was not significantly different—56.8 and 56.6 kg, respectively. Additionally, thirteen patients (30.2%) were diagnosed with obesity, defined as a body weight above the 95th percentile. Noteworthy is the fact that six patients (14%) reached the 100th percentile. A summary of additional information about the study group is provided in Table 1.

### 3.2. Effectiveness and Safety

At baseline, the mean PASI score was 16.3 ± 6.5, the mean body surface area affected by psoriasis was 22.3 ± 12.2%, and the mean CDLQI was 17.4 ± 5.3. Due to the severity of psoriatic lesions on the scalp, hands, and nails, three patients (7%) were qualified for etanercept therapy, despite not meeting the PASI, BSA, or CDLQI inclusion criteria.

**Figure 2:**
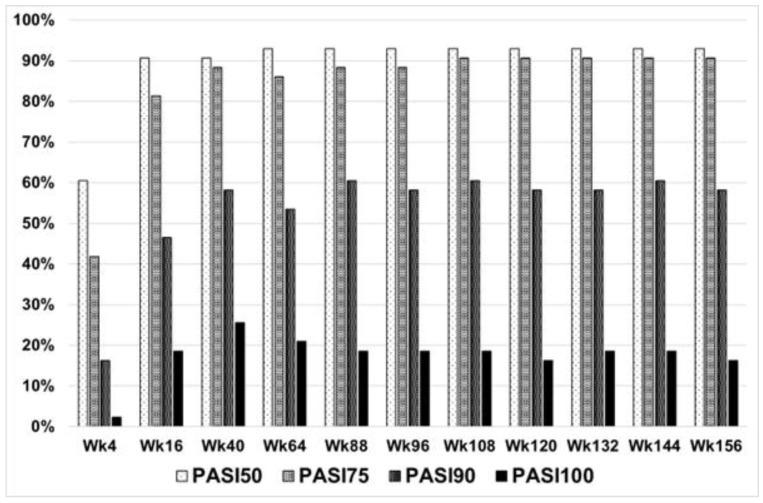
Percentage distribution of patients achieving PASI50, PASI75, and PASI90.

**Figure 3:**
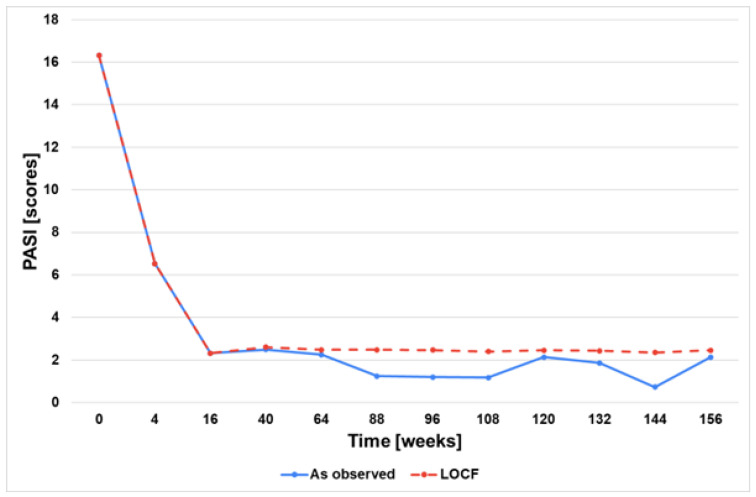
PASI scores during 156 weeks of etanercept therapy. LOCF—Last observation carried forward.

**Figure 4:**
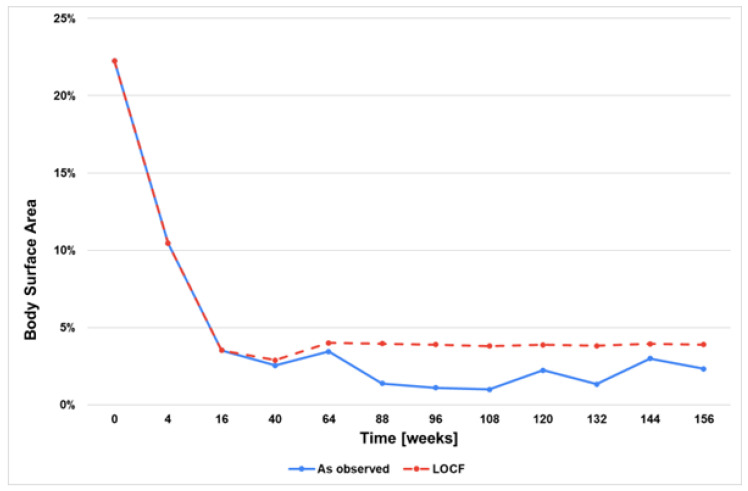
BSA scores during 156 weeks of etanercept therapy.

**Figure 5:**
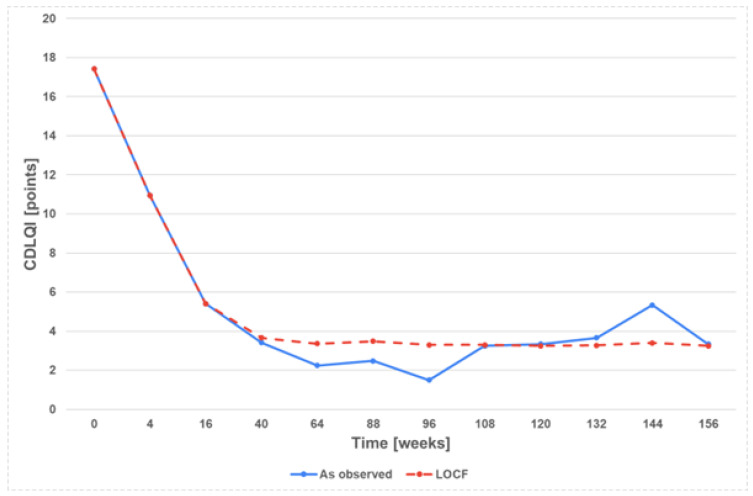
CDLQI points during 156 weeks of etanercept treatment.

**Figure 6:**
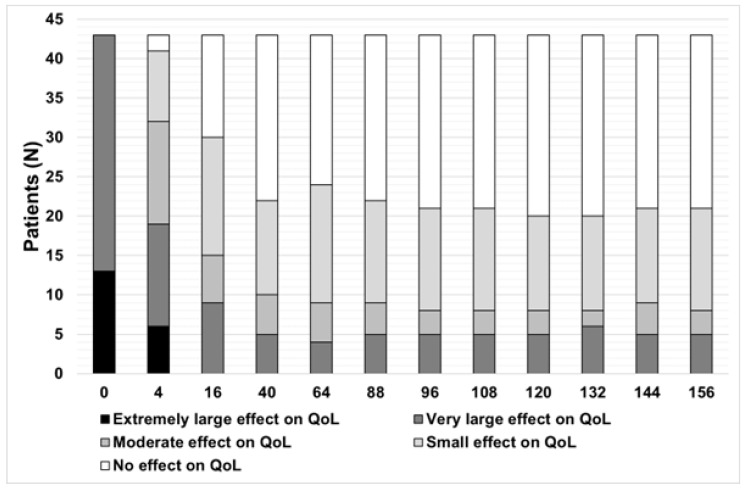
Impact of treatment on patients’ quality of life (QoL). Therapy had the largest effect during the first 16 weeks, after which the scores remained similar.

The mean PASI score, BSA, and CDLQI decreased to 6.5 ± 5.9, 10.5 ± 9.0%, and 10.9 ± 7.5, respectively, at week 4. However, the assessment of the treatment’s effectiveness was conducted at week 16. At that time, 90.7% (39 patients) achieved PASI 50, 79.1% (34 patients) achieved PASI 75, and 46.5% (20 patients) achieved PASI 90 (Figure 2). Similarly, the mean BSA and CDLQI scoring also significantly decreased at week 16 to the following values of 3.5 ± 3.8 and 5.4 ± 5.7, respectively. Additional data are summarised in Figure 2, Figure 3, Figure 4, Figure 5 and Figure 6. They show the satisfactory effects of etanercept treatment which were observed up to the 156th week of therapy, in the form of reductions in the PASI, BSA, and CDLQI scores.

The mean PASI score improvement during therapy varied according to the body weight percentiles on the growth chart. The patients classified as obese or overweight had a higher mean PASI score at baseline (17.32), week 4 (6.4), and week 16 (3.15) than the patients with healthy weight. In addition, only five out of thirteen obese patients (38.5%) achieved PASI 90, compared to ten out of fifteen (66.7%) patients in the 26–75 percentile range. The mean PASI improvement in the 95–100 percentile range was 80.47%, while in the 26–75 percentile range, it was 89.25%. However, no correlation between body weight and the response to the treatment in children representing different percentiles ranges was observed.

Regarding the safety, etanercept was well-tolerated, as there were neither any adverse events leading to treatment discontinuation, nor any adverse event considered as being related to the study drug.

## 4. Discussion

The results of our study validate etanercept as a safe and efficient medicine in moderate-to-severe plaque-type psoriasis in children, confirming earlier results from clinical trials [21,22] and observational studies [23,24,25,26,27,28] from other research teams at other research centres.

The fact that our study was conducted in accordance with the B.47 programme, where effectiveness endpoints were set at week 4, 16, 40, and 64, makes it difficult to accurately compare all the results of corresponding studies from other centres. Nevertheless, certain studies [23,29,30] in adults suggest that the onset of etanercept activity may be delayed in some patients; therefore, the usual 12-week endpoint is possibly too prompt to guarantee an accurate assessment of etanercept’s effectiveness. A significant difference was observed comparing our results from week 16 with those from other studies from week 12. At week 16, thirty-nine patients (90.7%) achieved PASI 50, thirty-four patients (79.1%) achieved PASI 75, and twenty patients (46.5%) achieved PASI 90. The other, one-year, retrospective, multi-centre study [28] investigating effectiveness, tolerability, and reasons for etanercept discontinuation in a real-life cohort of children and adolescents with moderate-to-severe plaque psoriasis reported a reduction in disease severity as measured by the PASI score (at week 12, 86.9% of patients achieved PASI 50, and 56.5% achieved PASI 75). In a 48-week, double-blind trial [15] of 12 once-weekly subcutaneous injections of a placebo or etanercept, at week 12, 75% patients receiving etanercept achieved PASI 50. PASI 75 and PASI 90, respectively, were achieved by 57% and 27% of patients. Our study confirms that the endpoint at week 16, with higher effectiveness results compared to week 12, is potentially better at assessing etanercept’s initial activity.

Patients received etanercept at a dose administered based on body weight with maximum dose of 50 mg subcutaneously once a week. No correlation between body mass and the treatment effectiveness among children representing different percentiles on the growth charts was observed; however, patients with obesity had a higher mean PASI score at baseline (17.32), week 4 (6.4), and week 16 (3.15) than patients with normal body weight. In this 48-week, randomised clinical trial [14], paediatric patients with psoriasis were treated with the same dosage of etanercept. Children who were treated with weight-based dosing had a better response than patients who received the maximum dose. Noteworthy is the fact that patients with the maximum dose of etanercept weighed more, were older, and had a longer history of psoriasis than those receiving lower doses. Consequently, a poorer response to the treatment in a certain profile of patients treated with doses smaller than 0.8 mg per kilogramme of body weight can be expected. In a Spanish, observational, retrospective study [23] including adult patients with plaque psoriasis, 19.6% of patients failed to respond to the usual dose of 50mg per week, and as a result, received an increased dose of 50 mg twice a week. After the mean of 24.1 ± 11.6 weeks, the 50 mg/wk dose was re-established, suggesting that in some patients, etanercept is efficient, though the maximum dosage of 50 mg is not sufficient. The uncertain data of the analysis of benefits of weight-based dosing without a determined maximum dose require further research.

No serious adverse events were observed in our study. During a major original randomised, controlled clinical study to evaluate the safety and effectiveness of etanercept in children with juvenile idiopathic arthritis [31,32,33], and in the subsequent eight years of follow-up, nine medically important infections were reported at a rate of 0.03 events per patient–year. The most common adverse effects (AEs) were injection-site reactions, upper respiratory tract infections, nasopharyngitis, and headache, which correspond to results from studies of psoriasis treatment with etanercept among children [15,34]. Moreover, the studies showed that repeated etanercept treatment, for up to eight years, is not associated with increased incidence of serious adverse events [31,32,33].

During the study time, etanercept was the only reimbursed treatment option for children in Poland. Since March 2023, we have had an extended variety of drugs within the B.47 programme, with an access to adalimumab in children above 4 years old and three more drugs in children over 6 years old, such as ustekinumab, ixekizumab, and secukinumab. Thanks to the availability of new therapeutic options, we can take pride in advancing the treatment of psoriasis among Polish children. Although etanercept proved to be an effective medicine, children, who failed to respond to that treatment, will have a chance to be treated with different therapeutic options, which makes it a promising subject for future research. It will also allow us to improve the treatment process of the most challenging cases.

Due to the nature of the study (real-life), not all patients reached the final observation periods. In order to fill in the missing data, two statistical analyses, ‘last observation carried forward’ and ‘as observed’, were used, which allowed the assessment of the effectiveness of the therapy over the entire follow-up period. Nevertheless, the fact that not all patients were observed for 156 weeks is an important limitation of the results obtained in the study.

## 5. Conclusions

This study confirmed the effectiveness of etanercept in reducing the severity of psoriasis over a period of 3 years in the paediatric population of thirty-seven children with moderate-to-severe plaque psoriasis. The etanercept treatment both significantly improved patients’ condition, reducing psoriatic symptoms but also positively affected their quality of life. It turned out to be safe for all children included in the study, with no considerable adverse events observed.

Psoriasis should be managed immediately after diagnosis. Biological drugs proved to be effective and safe in moderate-to-severe psoriasis; therefore, it is crucial to consider including them in the therapy

## Figures and Tables

**Table 1:** Characteristics of the patients (pts).

Number of Patients	43
Males	19
Females	24
Mean age of inclusion, (SD)	12.9 (3.1)
Range of age	6–less than 18 years old
Mean body weight, (SD)	61.8 kg (20.8)
Males—mean body weight (SD)	56.8 kg (22)
Females—mean body weight (SD)	56.6 kg (18.3)

## Data Availability

The data presented in this study are available on request from the corresponding author.
